# Fuzzy model to estimate the number of hospitalizations for asthma and pneumonia under the effects of air pollution

**DOI:** 10.1590/S1518-8787.2017051006501

**Published:** 2017-06-13

**Authors:** Luciano Eustáquio Chaves, Luiz Fernando Costa Nascimento, Paloma Maria Silva Rocha Rizol

**Affiliations:** I Departamento de Mecânica. Faculdade de Engenharia de Guaratinguetá. Universidade Estadual Paulista. São Paulo, SP, Brasil; II Fundação Universitária Vida Cristã. Faculdade de Pindamonhangaba. Pindamonhangaba, SP, Brasil; III Departamento de Medicina. Universidade de Taubaté. Taubaté, SP, Brasil; IV Departamento de Energia. Faculdade Engenharia de Guaratinguetá. Universidade Estadual Paulista. Guaratinguetá, SP, Brasil; V Departamento de Engenharia Elétrica. Faculdade de Engenharia de Guaratinguetá. Universidade Estadual Paulista. Guaratinguetá, SP, Brasil

**Keywords:** Air Pollution, adverse effects, Asthma, epidemiology, Pneumonia, epidemiology, Hospitalization, Fuzzy Logic

## Abstract

**OBJECTIVE:**

Predict the number of hospitalizations for asthma and pneumonia associated with exposure to air pollutants in the city of São José dos Campos, São Paulo State.

**METHODS:**

This is a computational model using fuzzy logic based on Mamdani’s inference method. For the fuzzification of the input variables of particulate matter, ozone, sulfur dioxide and apparent temperature, we considered two relevancy functions for each variable with the linguistic approach: good and bad. For the output variable number of hospitalizations for asthma and pneumonia, we considered five relevancy functions: very low, low, medium, high and very high. DATASUS was our source for the number of hospitalizations in the year 2007 and the result provided by the model was correlated with the actual data of hospitalization with lag from zero to two days. The accuracy of the model was estimated by the ROC curve for each pollutant and in those lags.

**RESULTS:**

In the year of 2007, 1,710 hospitalizations by pneumonia and asthma were recorded in São José dos Campos, State of São Paulo, with a daily average of 4.9 hospitalizations (SD = 2.9). The model output data showed positive and significant correlation (r = 0.38) with the actual data; the accuracies evaluated for the model were higher for sulfur dioxide in lag 0 and 2 and for particulate matter in lag 1.

**CONCLUSIONS:**

Fuzzy modeling proved accurate for the pollutant exposure effects and hospitalization for pneumonia and asthma approach.

## RESUMO

**OBJETIVO:**

Prever o número de internações por asma e pneumonia associadas à exposição a poluentes do ar no município em São José dos Campos, estado de São Paulo.

**MÉTODOS:**

Trata-se de um modelo computacional que utiliza a lógica *fuzzy* baseado na técnica de inferência de Mamdani. Para a fuzzificação das variáveis de entrada material particulado, ozônio, dióxido de enxofre e temperatura aparente foram consideradas duas funções de pertinência para cada variável com abordagem linguísticas: bom e ruim. Para a variável de saída número internações por asma e pneumonia, foram consideradas cinco funções de pertinências: muito baixo, baixo, médio, alto e muito alto. O número de internações no ano de 2007 foi obtido do Datasus e o resultado fornecido pelo modelo foi correlacionado com os dados reais de internação com defasagem (lag) de zero a dois dias. A acurácia do modelo foi estimada pela curva ROC para cada poluente e nestas defasagens.

**RESULTADOS:**

No ano de 2007 foram registradas 1.710 internações por pneumonia e asma em São José dos Campos, SP, com média diária de 4,9 internações (dp = 2,9). Os dados de saída do modelo mostraram correlação positiva e significativa (r = 0,38) com os dados reais; as acurácias avaliadas para o modelo foram maiores para o dióxido de enxofre nos lag 0 e 2 e para o material particulado no lag 1.

**CONCLUSÕES:**

Modelagem *fuzzy* se mostrou acurada para a abordagem de efeitos da exposição aos poluentes e internação por pneumonia e asma.

## INTRODUCTION

Air pollution is a serious environmental issue, given its impact on human health, especially cardiovascular and respiratory systems^1^ ^,^ ^2^.

In Brazil, especially in large urban centers, exposure to pollutants like particulate matter (PM_10_), sulfur dioxide (SO_2_), ozone (O3), carbon monoxide (CO) and nitrogen oxides (NOx) is associated with hospitalizations for respiratory diseases such as asthma and pneumonia, thanks to the pollutants easy reach to the respiratory tract^3^ ^,^ ^16^.

Currently, this issue extends beyond major urban centers^7^ ^,^ ^11^ and is affecting cities of medium and small size^1^ ^,^ ^6^ ^,^ ^8^. Exposure to air pollutants represents the high financial cost for the public network. The cost of 900,000 hospitalizations for pneumonia and asthma in 2011 reached US$350 million in Brazil, US$70 million in São Paulo State, with 150,000 hospitalizations, and US$800,000 in the city of São José dos Campos, with 1,900 hospitalizations^a^, representing a public health problem.

The statistical techniques of logistic regression and Poisson’s regression (Generalized Linear Models or Generalized Additive Models) are often used to estimate the chance or risk of hospitalization or death for respiratory diseases^9^.

A new form of epidemiological data analysis in public health is fuzzy logic. The fuzzy set theory was introduced by Lotfi A. Zadeh^23^, in 1965, and can work with the vague aspect of information particular to human understanding and very common in the medical field, in which descriptions of diseases often comprise language terms that are inevitably vague, such as fever (high or low)^15^. Unlike the classical set theory, in which an element belongs or does not belong to a set, in the theory of fuzzy sets an element may belong to more than one set with different degrees of relevance (varying between zero and one). These fuzzy sets are represented by the relevancy function, whose determination depends on the specialist’s individual perception and the data at hand^17^.

The ability to deal with linguistic terms can explain the increase in the number of studies that use fuzzy logic in biomedicine problems. In fact, the fuzzy logic theory has become an important approach in diagnosis systems, prognosis, forecasting models, medical treatment and, more recently, in epidemiology and public health^13^ ^,^ ^15^ ^,^ ^17^ ^,^ ^18^ ^,^ ^20^ ^,^ ^22^ ^,^ ^23^ ^,^ ^b^ *.*

This study’s objective was to develop a computational model using fuzzy logic to estimate the influence of exposure to air pollutants in the number of hospital admissions for asthma and pneumonia.

## METHODS

This is a computational model using fuzzy logic to estimate the number of hospitalizations for pneumonia and bronchial asthma per the concentrations of the pollutants particulate matter (PM_10_), ozone (O_3_) and sulfur dioxide (SO_2_) and the apparent temperature (TEMPap), calculated by considering ambient temperature and relative air humidity^2^.

The environmental pollutants and climatic variables data in this study were obtained from the Environmental Company of the State of São Paulo (CETESB), which has a measuring station in the city of São José dos Campos. The data of the number of hospitalizations for pneumonia and bronchial asthma (ICD-10: J12 to J18 and J45) in individuals of all ages residing in São José dos Campos, in the period from 1/1/2007 to 12/31/2007, were obtained from the Department of Information and Computer Science of the Brazilian Unified Health System (DATASUS).

This study was carried out in São José dos Campos, a medium-sized city in the State of São Paulo, which has an important industrial park. It is located at 23°10’S and 45°52’O, in the Alto Vale do Paraíba, 600 m above sea level, has wet weather and tropical altitude, located between São Paulo and Rio de Janeiro – the two largest cities in Brazil; is cut through by the Via Dutra, the most important highway in the country with heavy traffic of buses and trucks. Its population is estimated to be around 650,000 inhabitants.

The model was developed with the help of a specialist who created two functions of relevance for the input variables: PM_10_ – good and bad; O_3_ – good and bad; SO_2_ – good and bad; and TEMPap – good and bad. These fuzzy sets were drawn based on the data obtained from CETESB^c^.

The output variable was the number of hospitalizations for asthma and pneumonia and its five relevance functions we classified as follows: very low, low, medium, high and very high.

The fuzzy linguistic model is a rule-based inference system using fuzzy set theory to address the phenomenon. Its structure includes four components:

- The fuzzifier, which transforms real inputs (also known as crisp) into fuzzy values;
- The rule base, which defines the connection between the system’s inputs and outputs. A fuzzy rule has the following form: if precedent, then consequent, in which the precedent can be composed of one or more fuzzy sets connected by fuzzy operators. And the consequent represents the fuzzy values of output variables;
- The inference system, which evaluates all rules, checks which have been activated (with degree of relevance greater than zero) and combines the resulting weights of all the rules enabled on a single output (in this study, we used the Mamdani’s inference system); and
- The defuzzifier that performs the reverse process of the fuzzifier, that is, it transforms the fuzzy output into an actual value (crisp)^21^.

When performing the combination of all possible entries, it was possible to develop 16 rules resulting from the combination of the four entries with two functions of relevance each (2 × 2 × 2 × 2), drawn up with the aid of an expert. For example, two combinations could be:

IF PM_10_ IS GOOD AND O_3_ IS GOOD AND SO_2_ IS GOOD AND TEMPAP IS GOOD THEN THE NUMBER OF HOSPITALIZATIONS IS VERY LOW

IF PM_10_ IS BAD AND O_3_ IS BAD AND SO_2_ IS BAD AND TEMPAP IS BAD THEN THE NUMBER OF HOSPITALIZATIONS IS VERY HIGH

The number of hospitalizations of the fuzzy linguistic model was determined by the inference method (fuzzy) proposed by Mamdani^23^, which consists in calculating the minimum (degrees of activation) of the rule’s precedents and, subsequently, the aggregation of the rule’s consequents (maximum operator). Finally, the defuzzification is performed, based on the area center method^17^.

Through a routine (toolbox fuzzy) that is part of the program MATLAB®^21^, we obtained the numerical output resulting from the fuzzy model, providing the number of hospitalizations for each combination of the database entry. After obtaining the model’s result, we performed a Pearson correlation with actual admissions data, using up to two days of lag (lag 2) because the display effect can manifest on the same day (lag 0) or on subsequent days. The accuracy, along with its 95% confidence interval was estimated using the ROC curve.

Because the data are available on the network and cannot be identified, a submission to the Research Ethics Committee was unnecessary.

## RESULTS

In the period study, 1 January, 2007 to 31 December, 2007, 1,710 hospitalizations were recorded according to DATASUS^a^.

Table 1 shows the mean value, standard deviation, the minimum and maximum value of the PM_10_, O_3_, SO_2_, TEMPap variables and the number of hospitalizations.

**Table 1:** Values of the averages, standard deviation (SD), minimum and maximum of the variables particulate matter (PM10), ozone (O3), sulfur dioxide (SO2), apparent temperature (TEMPap) and the number of hospitalizations (NINTER). São José dos Campos, State of São Paulo, Brazil, 2007.

Input/output variables	Average	SD	Minimum	Maximum
PM_10_ (μg/m^3^)	26.0	11.3	8.0	89.0
O_3_ (μg/m^3^)	91.0	67.9	17.0	162.0
SO_2_ (μg/m^3^)	3.8	3.3	0.9	27.0
TEMPap (°C)	17.9	0.9	5.7	20.2
NINTER	4.9	2.9	0	16.0

Twelve times ozone exceeded the value used by the National Council for the Environment (CONAMA)^d^. Particulate matter and sulfur dioxide did not exceed those limits.

**Figure 1:**
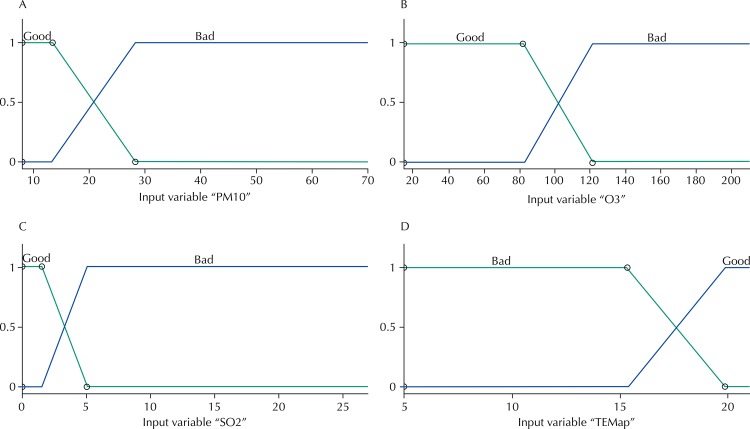
Input function of relevance: (A) Particulate matter (PM10), (B) Ozone (O3), (C) sulfur dioxide (SO2) and (D) apparent temperature (TEMPap), of the fuzzy model for estimation of the number of hospitalizations in the city of São José dos Campos, State of São Paulo, Brazil, 2007.

**Figure 2:**
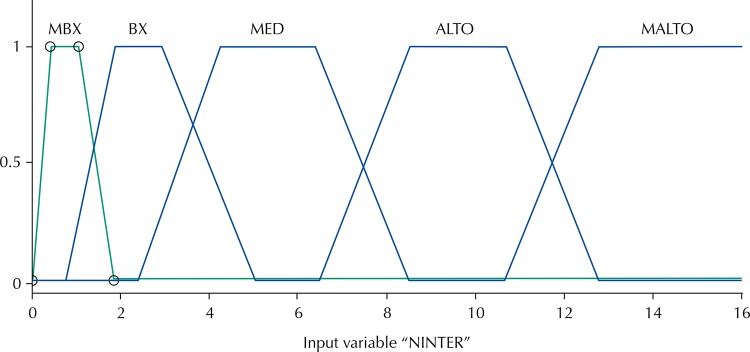
Output functions of relevance (number of hospitalizations) very low (MBX), low (BX), medium (MED), alto (ALTO) and very high (MALTO) for the fuzzy model in the city of São José dos Campos, State of São Paulo, Brazil, 2007.

Relevancy functions of input variables: PM_10_; O_3_; SO_2_ and TEMPap are shown in Figure 1 (A-D). The output variable: number of hospitalizations for asthma and pneumonia with its five relevancy functions are shown in Figure 2.

The results obtained by the ROC curve are presented in Table 2. The best result was obtained for the zero-day lag (lag 0), PM_10_ and SO_2_ showed the best performance per the ROC curve, with statistically significant values.

**Table 2:** Roc curve values for lag 0, lag 1 and lag 2 of the pollutants PM10, O3, and SO2, per output type, the number of hospitalizations up to 2. São José dos Campos, State of São Paulo, Brazil, 2007.

Input variable	*Lag* 0 (95%CI)	*Lag* 1 (95%CI)	*Lag* 2 (95%CI)
PM_10_	0.92 (0.88–0.96)	0.75 (0.64–0.85)	0.71 (0.60–0.81)
O_3_	0.87 (0.82–0.92)	0.64 (0.52–0.76)	0.62 (0.51–0.73)
SO_2_	0.95 (0.92–0.97)	0.73 (0.63–0.82)	0.79 (0.72–0.87)

The Pearson correlation coefficient between the output of the fuzzy model and the actual data were 0.38 for lag 0; 0.36 for lag 1 and 0.30 for lag 2 (p < 0.01); even though the values are not high, they were significant.

## DISCUSSION

This article presents the development of a computational model using fuzzy logic to estimate the number of hospitalizations for asthma and pneumonia associated with air pollutants in a midsize industrial town. The results showed good accuracy when predicting the number of hospitalizations when exposure occurred on the same day and up to two days later. Additionally, we saw the acute effect the exposure to pollutants has in hospitalizations.

This study showed a maximum concentration of PM_10_ of 89 μg/m^3^, which is lower than the air quality advocated by CETESB^c^, which is 120 μg/m^3^. According to CETESB, PM_10_ indexes between 51 and 100 μg/m^3^ classify an air of moderate quality and can cause symptoms such as a dry cough and fatigue in patients with respiratory diseases.

In the study by Arbex et al.^2^, PM_10_ may lead to airway irritation, inflammation, increased bronchial reactivity and decreased mucociliary activity, and its consequences are an increase in asthma attacks and respiratory infections.

In research conducted by Gouveia et al.^12^ in the city of São Paulo, State of São Paulo, PM_10_ presented an average of 54.5 μg/m^3^, a value well above this study’s average, which was 26 μg/m^3^. We found that, with a 10 μg/m^3^ increase in the concentration of particulate matter, there was an increase of approximately 5% in the number of hospitalizations for asthma in children. Similarly, Nascimento et al.^16^ observed that, with an increase of 24.7 μg/m^3^ on the average concentration of PM_10_, there was an increase of 9.8% in hospitalizations for pneumonia in children in the city of São José dos Campos, with an average PM_10_ concentration of 40 μg/m^3^.

In the study performed with data from 2004 and 2005 in the same city^1^, the average was 25.2 μg/m^3^, a value close to the one found in this study, and hospitalizations for asthma were associated with exposure to PM^10^.

As for O_3_, the average value found in this study was o 91 μg/m^3^ for the period of 2007, close to the value found by Amâncio and Nascimento^1^ in São José dos Campos with an average of 74.3 μg/m^3^, by Gouveia et al.^12^, with an average of 71.8 μg/m^3^, and greater than the one observed by Negrisoli and Nascimento^19^, with an average of 37.1 μg/m^3^. On 12 days, the O_3_ exceeded the values of acceptable levels (up to 160 μg/m^3^) at CONAMA^d^. In CETESB’s^c^ report, the inappropriate values for O_3_ are between 180μg/m^3^ and 200 μg/m^3^ and can aggravate asthma symptoms in children with respiratory disease. In the general population, it can lead to the following symptoms: a dry cough, fatigue and burning sensation in the nose, throat, and eyes. The concentration of O_3_ had a significant growth in the last year in the city of São José dos Campos, per CETESB’s latest report^c^.

The concentration of SO_2_ had an average of 3.8 μg/m^3^, which is within the values tolerated by the World Health Organization (WHO), which accepts a maximum exposure level of up to 20 μg/m^3^ for 24 hours. This value was like the one found previously (4.6 μg/m^3^) in the same city^1^, with data collected between 2004 and 2005, and another study, also in São José dos Campos, averaging 6.2 μg/m^3^, with data from 2000 and 2001^16^.

In a research conducted in the city of São Paulo, Gouveia and Fletcher^10^ found an average concentration of SO_2_ of 17.71 μg/m^3^, which differs from the value found in this study. This difference can be explained by the larger fleet of vehicles in São Paulo, a major source of this pollutant.

The pollutants cited in this article and their magnitudes are described in national articles about this subject^1^ ^-^ ^7^ and it is important to note that the data of hospital admissions are related to hospitalization in the public health system. Even at concentrations considered safe, the pollutants can cause adverse health effects, especially on the cardiovascular and respiratory systems.

This study differs from other studies that estimate risks or chances of hospitalizations caused by exposure to air pollutants using logistic regression, retrospective studies or Poisson’s regression^1^ ^,^ ^3^ ^,^ ^5^ ^,^ ^6^. In these models, fuzzy logic has the advantage of dealing with the uncertainty of information present in the meanings of words, facilitating dialogue between health care professionals and computational experts.

The fuzzy model showed itself to be very satisfactory by associating exposure to pollutants with the number of hospitalizations when compared with real outputs, with Pearson correlation coefficient of 0.38. This methodology was used recently in the same city to analyze the average time of hospitalization arising from pneumonia^18^. In the national literature, there are also articles with fuzzy application in establishing the risk of neonatal death^15^ ^,^ ^17^ and neonatal resuscitation prediction^20^.

In epidemiological studies, it is common to find an association between the concentrations of air pollutants and the health effects on the next day, after two days or even after a week. Researchers generally adjust the model for different arrangements of the same database with lags. In time-series studies, lags of one to seven days are often used^4^ ^,^ ^14^.

The data obtained by the ROC curve of the model (Table 2) showed excellent accuracy and PM_10_, O_3_, and SO_2_ had a good performance with lag zero, which allows us to predict the effects of these pollutants on the same day that exposure occurs. The best result was for the pollutant SO_2_ at zero lag, with great accuracy and area under the curve of 95%. Thus, we can conclude that the more PM_10_ and SO_2_ are present in the atmosphere, the more hospitalizations of patients with asthma and pneumonia will occur.

The actual data regarding the number of hospitalizations ranged from zero to 16 and those obtained by the fuzzy model ranged from 0.7 to 13.9. This difference between minimum and maximum is due to the defuzzification method of the fuzzy inference system, in that the result is obtained by calculating the area center. Therefore, it would be mathematically difficult to obtain the area center around the ends of the universe of discourse of the output variable.

To improve the model, we can include more relevance functions in the input variables, which would involve a larger number of rules; or use different formats of relevance functions, such as Gaussian or triangular. The concentrations of pollutants are considered homogeneous for the implementation of this approach, which can be considered a limitation, since concentrations in other areas of the same city may differ from those recorded near the monitoring station.

This research is a low-cost financial tool and can be presented in a specific computer program (expert system) for this purpose, and does not require the opinion of experts. The model can be implemented in public health systems and can serve as an important instrument for prevention and decision-making regarding changes in the level of pollutants. It can also be applied in any locations where there are available data on pollutants and climatic conditions.

## Footnotes

a Ministério da Saúde (BR), Departamento de Informática do Sistema Único de Saúde – Datasus. Brasília (DF); c2008 [cited 2012 Aug 12]. Available from: http://www2.datasus.gov.br/DATASUS/index.php

b Ortega NRS. Aplicação da Teoria dos Conjuntos Fuzzy a problemas de biomedicina [these]. São Paulo (SP): Instituto de Física da Universidade de São Paulo; 2001.

c CETESB - Companhia Ambiental do Estado de São Paulo. Sistema Qualar. São Paulo; [cited 2017 Mar 20]. Available from: http://ar.cetesb.sp.gov.br/qualar/

d Ministério do Meio Ambiente (BR). Resolução Conama nº 003, de 28 de junho de 1990. *Diario Oficial Uniao*. 22 ago 1990 [cited 2012 Aug 10]; Seção 1:15937-9. Available from: http://www.mma.gov.br/port/conama/res/res90/res0390.html
